# GERONTOLOGICAL BIOSTATISTICS AS THE FOUNDATION OF INTERDISCIPLINARY DATA SCIENCE IN AGING

**DOI:** 10.1093/geroni/igac059.709

**Published:** 2022-12-20

**Authors:** Thomas Travison

**Affiliations:** Harvard Medical School, Boston, Massachusetts, United States

## Abstract

Explosive growth in computing power has increased by orders of magnitude the complexity of data structures and the number of analyses that may be performed per unit time. Contemporary gerontology utilizes diverse data ranging from continuous longitudinal assessments (e.g. motion capture) to complex single-timepoint assessments (e.g. bioimages) to systems-level administrative descriptions of the healthcare delivery environment. Paradoxically, this abundance of resources presents a considerable challenge, as the availability of information threatens to overwhelm mechanistic models of aging supportive of intervention development. To inform precision medicine, gerontological biostatistics therefore embraces the opportunity of collaborating with allied quantitative disciplines to bolster the coherence, reproducibility, and generalizability of findings. This presentation will demonstrate the salient advantages of such interdisciplinary collaborations using the example of design and analysis of an intensely longitudinal study of wearable and environmental sensors, conducted with teams in exercise science and architectural design.

